# Lifespan Predicts Mitochondrial Substitution Rates across Vertebrates, but Methodology Matters

**DOI:** 10.1093/gbe/evag067

**Published:** 2026-03-16

**Authors:** Jess E Sterling, Kendra D Zwonitzer, Justin C Havird

**Affiliations:** Department of Molecular Biosciences, The University of Texas at Austin, Austin, TX, USA; Department of Molecular Biosciences, The University of Texas at Austin, Austin, TX, USA; Department of Molecular Biosciences, The University of Texas at Austin, Austin, TX, USA; Department of Integrative Biology, The University of Texas at Austin, Austin, TX, USA

**Keywords:** mitochondrial DNA, substitution rates, generation time, longevity, comparative genomics, phylogenetic comparative methods

## Abstract

Why do some species live for mere months, while others persist for centuries? A leading explanation implicates mitochondria. The mitochondrial theory of aging predicts that mitochondrial efficiency diminishes with age due to the accumulation of mutations within mitochondrial DNA (mtDNA). While experimental evidence for this theory is mixed, evolutionary analyses offer an ideal opportunity to determine if mitochondrial substitution rates are linked to longevity. Here, we explored the relationship between mtDNA evolution and species' lifespans across four clades—Aves, Actinopterygii, Bivalvia, and Sebastidae—using five normalization strategies. Across most methods, long-lived vertebrates showed reduced synonymous and nonsynonymous substitution rates, suggesting lower mtDNA mutation. However, we found that the strength and direction of these relationships varied drastically depending on the normalization approach used (ie correcting for divergence, generation time, and phylogeny). We also analyzed mtDNA mutation spectra and found similar patterns in long- and short-lived species, suggesting decreased rates of mtDNA mutations in long-lived species are not due to suppression of specific mutation processes, as predicted from the free radical theory of aging. We also find little evidence for a relationship between selection on mitochondrial protein-coding genes and lifespan. Our results align with the idea that decreased mutation rates may help preserve mitochondrial integrity in long-lived vertebrate species, but that these species have not been selected to have particularly efficient OXPHOS or protection against a specific mitochondrial mutation process. Together, these findings underscore the critical link between mitochondrial stability and lifespan, and highlight the power of natural systems in this field.

Significance Comparative studies often report that long-lived species have lower mtDNA substitution rates, supporting mitochondrial explanations for aging. We show that many of these conclusions rely on how rates are normalized, especially with respect to generation time. Using four animal clades and multiple analytical approaches, the lifespan–rate association largely dissolves under appropriate controls. An informative exception arises in rockfish, where longer-lived species accumulate fewer substitutions per generation, aligning with life histories in which reproductive success increases with age. By combining genome-wide estimates, gene-level analyses, and mutation spectra, we clarify what aspects of mitochondrial evolution truly track longevity. Our work provides a practical framework for future studies, demonstrating that methodological rigor is essential for separating robust biological signals from artifacts.

## Introduction

From the ephemeral lives of mayflies to the centuries-old bowhead whales, the diversity of lifespans across species raises a fundamental question: What controls the rate of aging? One consistent pattern that persists across aging is that, as organisms age, critical components of biological processes and structures that sustain life begin to degrade. From a genetic perspective, this degradation is evident from the rapid accumulation of deleterious mutations in old age (Medawar 1952; Rose 1994; Turan et al. 2019; Ren et al. 2022). This accumulation can be explained in an evolutionary context by the gradual weakening of natural selection beyond reproductive age (Medawar 1952; Williams 1957; Hamilton 1966; Rose 1994; Austad and Hoffman 2018). Mutations that are slightly deleterious in early age but highly deleterious in old age can drift to fixation, impacting age-associated phenotypes (Rose and Charlesworth 1980; Maklakov and Chapman 2019). In a similar vein, antagonistic pleiotropy predicts that alleles that are beneficial in the early stages of life will be selected for, even if they are detrimental in old age (Williams 1957; Maklakov and Lummaa 2013; Austad and Hoffman 2018; Long and Zhang 2023). However, the proximate factors that drive the physiological degradation of animals in late age are still an open area of investigation. Although it is highly probable that aging is shaped by evolutionary trade-offs favoring early-life success, the molecular mechanisms and specific genes underlying these evolutionary patterns are still of great interest.

Mitochondria have emerged as likely key players connecting evolutionary theories of aging to cellular damage. Mitochondria, through oxidative phosphorylation (OXPHOS), produce both life-sustaining energy in the form of ATP (Alberts et al. 2002) and highly destructive molecules known as reactive oxygen species (ROS). A large body of research links mitochondrial decline to hallmark features of aging, including reduced cellular energy production (Trounce et al. 1989) and increased oxidative stress (Wallace 2001). Over the course of a lifespan, organisms also experience an age-dependent increase in somatic mtDNA mutations (Kennedy et al. 2013; Z. Wang et al. 2025). This mitochondrial mutation accumulation in somatic tissues is directly correlated with increased mitochondrial dysfunction and may be relevant to the shortening of an organism's life as predicted from mutation accumulation theory, which concerns mutations in the germline. Although somatic mtDNA mutation accumulation within individuals is conceptually distinct from germline mutation fixation across generations, recent comparative work suggests that somatic and germline mutation rates may nonetheless covary across taxa (G. Wang et al. 2025). While the rate of mtDNA mutation accumulation varies drastically from species to species, the pattern is relatively consistent within the species that have been investigated (Nabholz et al. 2007). As a result, species with shorter maximum lifespans experience a significantly faster accumulation of age-related mtDNA mutations compared to long-lived species (Khaidakov et al. 2003; Nabholz et al. 2007). Moreover, ROS have been hypothesized to be the driving force behind cellular degradation (Harman 1956). In this theory, ROS damage to mtDNA accumulates over time, impairing function and thus accelerating aging and the accumulation of more mtDNA mutations and more ROS in a vicious cycle (Harman 1972). Coined the mitochondrial free radical theory of aging (MFRTA), this idea has been a dominating model for mitochondrial aging for decades (Harman 1956; Harman 1972; Sanz and Stefanatos 2008; Bratic and Larsson 2013; Lagouge and Larsson 2013).

However, mounting evidence has cast doubt on the MFRTA. Recent studies show that the majority of mtDNA mutations are not caused by ROS (Kujoth et al. 2005; Lapointe and Hekimi 2010) but may derive from mtDNA replication errors (Hood et al. 2019). The broader debate on the role of mtDNA mutations in aging is ongoing, as some studies continue to highlight the potential consequences of mtDNA mutations for aging and disease, while others argue that these mutations do not significantly limit lifespan (Vermulst et al. 2007; Smith et al. 2020; Wolf 2021). For example, the PolG mutator mice accumulate mtDNA mutations via a defective mtDNA polymerase and also age rapidly, although littermates that inherit the same mtDNA mutations do not age rapidly (Kujoth et al. 2005). The collective results suggest the existence of a more complex dance between mitochondria and aging.

Is there a single, ultimate source of mtDNA mutations, and how might some species resist its effects to have prolonged lifespans? To answer this, two primary approaches have been used. First, transgenic, mutant models allow observation of mitochondrial aging in controlled settings to directly link mtDNA mutations with changes in lifespan. Some models do suggest that increasing mtDNA mutation rates through mtDNA polymerases with defunct proofreading shortens lifespan, while reducing mutations extends it (Trifunovic et al. 2004; Yamamoto et al. 2005; Taguchi et al. 2007). However, these studies reveal little about the natural mechanisms behind longevity in the wild, and only a limited number of model species have been explicitly investigated.

A second powerful approach is the use of comparative phylogenetic analyses that can take advantage of the extreme variation in lifespan observed across the tree of life. Such studies can link natural variation in lifespan to protection against sources of degradation and mutation accumulation in mtDNA. Past comparative phylogenetic studies have shown that long-lived species consistently possess lower mtDNA substitution rates across diverse animal clades, including mammals (Nabholz et al. 2007), birds (Nabholz et al. 2009), rockfishes (Kolora et al. 2021), and bivalves (Mortz et al. 2021). This suggests a possible link between longevity and reduced mtDNA mutation rates, potentially reflecting protective mechanisms against ROS or replication errors in long-lived species. However, most studies have focused on single genes and do not examine substitution patterns across the full mitochondrial genome. Moreover, few, if any, studies have analyzed substitution spectra, which could indicate the mutational processes involved. For instance, a higher proportion of G→T substitutions in short-lived species may suggest more oxidative damage (Hahm et al. 2022), lending support to the MFRTA.

In this study, we take a comparative approach within four taxonomic groups (Aves, Actinopterygii, Bivalvia, and Sebastidae) by (i) analyzing mitochondrial protein-coding sequences (CDS) across the full mitogenome in species with varying lifespans to assess substitution rate patterns and gene-specific effects, (ii) characterizing substitution spectra to infer mutational processes across species with different lifespans, and (iii) testing whether selection on mtDNA varies with lifespan. We also assessed how different normalization methods affected our results. Under the MFRTA, we hypothesized that longer-lived species would have lower mtDNA substitution rates and a lower proportion of G→T substitutions associated with ROS damage (especially dS, which should reflect mutation rates more directly).

## Results

### Long-Lived Species Tend to Have Lower mtDNA Substitution Rates in Vertebrates

To assess how normalization methodology impacts the relationship between maximum lifespan and mtDNA substitution rates, we used five normalization approaches: unnormalized (raw rates), normalized for divergence time, normalized for generation time, and normalized for the latter two with phylogenetic correction. Raw substitution rates reflect observed sequence divergence, whereas divergence time normalization estimates evolutionary rates per unit time, and generation time normalization scales substitution rates by reproductive turnover, a biologically relevant proxy for germline mutational opportunity. Phylogenetically corrected models further account for nonindependence among species due to shared evolutionary history. Under divergence time normalization, we expected substitution rates to primarily reflect time-dependent mutation accumulation, predicting lower rates in long-lived species if lifespan is associated with reduced mutational input. In contrast, generation time normalization tests whether differences in substitution rates are better explained by reproductive turnover, in which case accounting for generation time could weaken or reverse lifespan–rate relationships.

**Fig. 1.**
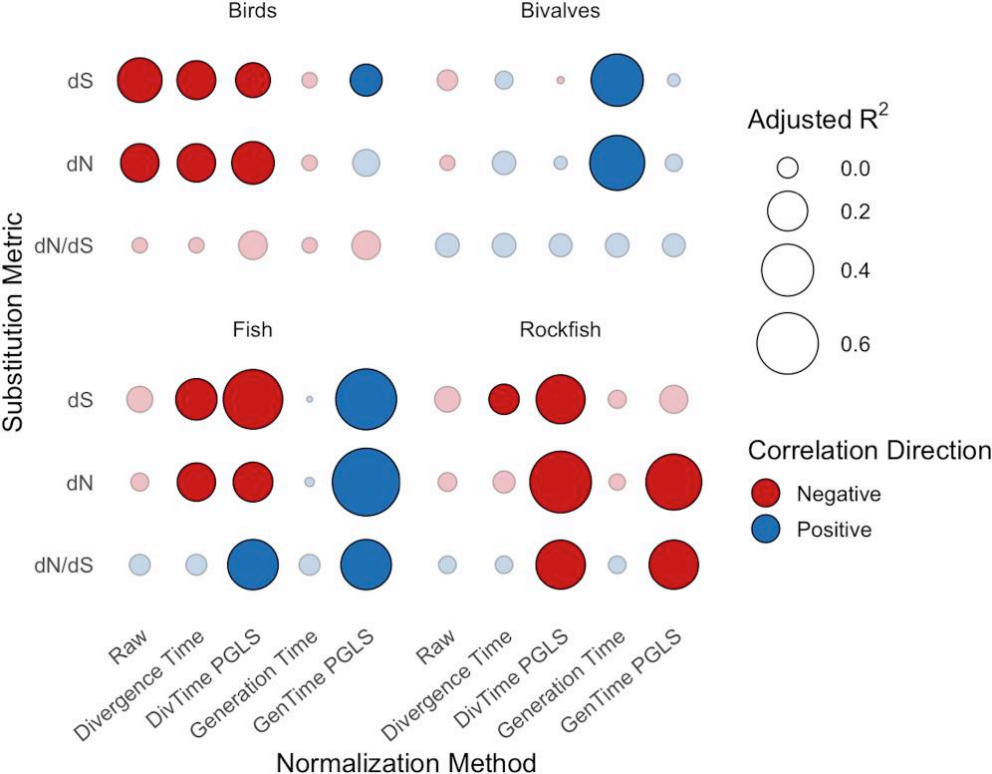
Effect of normalization method on the strength and direction of max lifespan–substitution rate correlations across clades. Bubble plot showing the adjusted *r*^2^ values for correlations between maximum lifespan and mitochondrial substitution metrics (dN, dS, dN/dS) across normalization methods and clades. Point color indicates the direction of the correlation (blue, positive; red, negative), while point transparency reflects statistical significance (opaque, *P* < 0.05; translucent, nonsignificant). Bubbles increase in size with higher explanatory power (*r*^2^). Rates were normalized to divergence time, generation time, and/or corrected for phylogeny (PGLS). The dN/dS ratio was not normalized by divergence or generation time, so values are identical within PGLS and non-PGLS models, differing only by phylogenetic correction. All *r*^2^ and *P*-values were calculated by regressing log-transformed substitution metrics against log-transformed maximum lifespan, using standard linear models for non-PGLS normalization methods and phylogenetic generalized least squares (PGLS) for the PGLS-based methods.

Across this framework, we evaluated the strength (*r*^2^) and direction (slope sign) of correlations between dN and dS and lifespan and across clades (Fig. 1). Across vertebrate clades (birds, fishes, and rockfishes), nearly all normalization methods led to a negative correlation between lifespan and both synonymous and nonsynonymous substitution rates (Fig. 1). The primary exception was the generation time–normalized phylogenetic model [GenTime + phylogenetic generalized least squares {PGLS}], which produced a significant positive correlation between lifespan and mtDNA substitution rates in birds and fishes (Fig. 1). While many of the normalization strategies yielded similar trends, the divergence time + PGLS and generation time + PGLS models consistently showed the highest explanatory power and statistical support. Because these two approaches reflect contrasting biological interpretations of mtDNA substitution rate variation, below we focus on these two representative models.

**Fig. 2.**
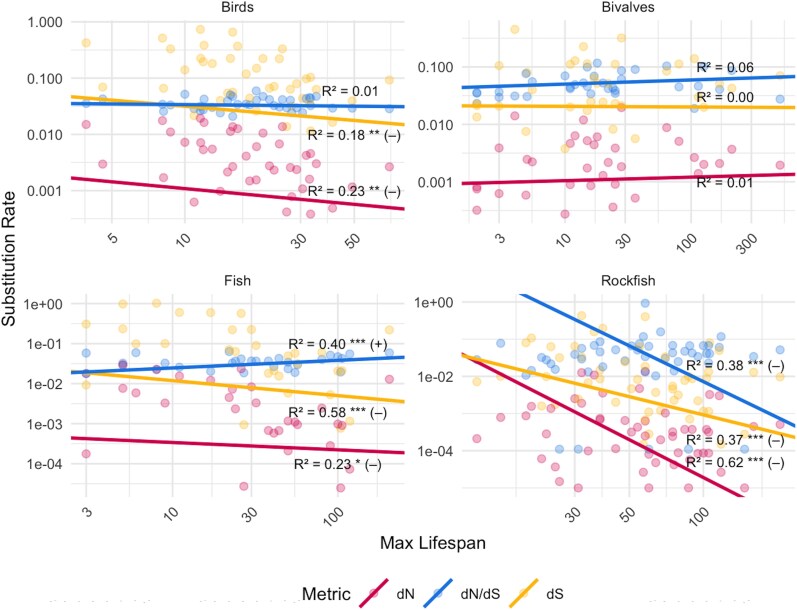
Correlation between max lifespan and dN, dS, and dN/dS across four clades. Relationships between maximum lifespan (*x* axis, log scale) and three mtDNA rates of evolution (*y* axis, log scale): dN, dS, and dN/dS for three datasets (birds, fish, rockfish, and bivalves). dN and dS are corrected for phylogeny and divergence times (eg units on the *y* axis are dN or dS per million years divergence time). dN/dS is only corrected for phylogeny using PGLS. Solid lines represent PGLS regression trends (phylogenetically corrected), with associated *r*^2^ values shown. Direction of trend (+ or −) and statistical significance are also shown (*P < 0.05, **P < 0.01, and ***P < 0.001).

Correlative analyses between mtDNA substitution rates and lifespan corrected for divergence times and phylogeny showed a negative correlation across all vertebrate datasets (Fig. 2). This was statistically significant for both nonsynonymous and synonymous substitution rates for birds (*P* ≤ 0.007, *r*^2^ ≥ 0.17 for both dN and dS), fish (*P* ≤ 0.019, *r*^2^ ≥ 0.22 for both dN and dS), and rockfish (*P* ≤ 0.001, *r*^2^ ≥ 0.36 for both dN and dS) datasets based on PGLS correlations between maximum lifespan and substitution rates corrected for divergence times (Fig. 2a, c, and d). To contextualize the effect sizes, a projected 10-fold increase in maximum lifespan was associated with a ∼35% to 70% reduction in dN and a ∼60% reduction in dS within the fish and bird clades (Table S1). In the one invertebrate clade, bivalves, nonsignificant positive trends were observed for both dN and dS (*P* ≥ 0.616, *r*^2^ ≤ 0.02 for both) (Fig. 2b).

Using the generation time + PGLS normalization method (dN and dS per 100,000 generations, accounting for both generation time and divergence time, and corrected for phylogeny), the trends were very different from the divergence time–normalized values (Fig. 1 and 2, Fig. S1). The exception being the rockfish clade, which retained its negative correlation with max lifespan for dN and dS, but only dN remained statistically significant (*p*^dN^ = <0.001, *r*^2(dN)^ = 0.50, *p*^dS^ = 0.112, *r*^2(dN)^ = 0.09). For the bird and fish clades, the previously observed trends flipped when calculating rates per 100,000 generations: A significant positive correlation between dN and dS with max lifespan was observed in both the bird (*P* ≤ 0.047, *r*^2^ ≥ 0.10 for both dN and dS) and fish (*P* ≤ 0.001, *r*^2^ ≥ 0.60 for both dN and dS) clades when using generation times. For bivalves, the correlation with max lifespan remained statistically nonsignificant (*P* ≥ 0.444, *r*^2^ ≤ 0.03 for both dN and dS). All relevant PGLS statistics (*P*-value, slope, intercept, and *r*^2^) can be found in Table S1.

**Fig. 3.**
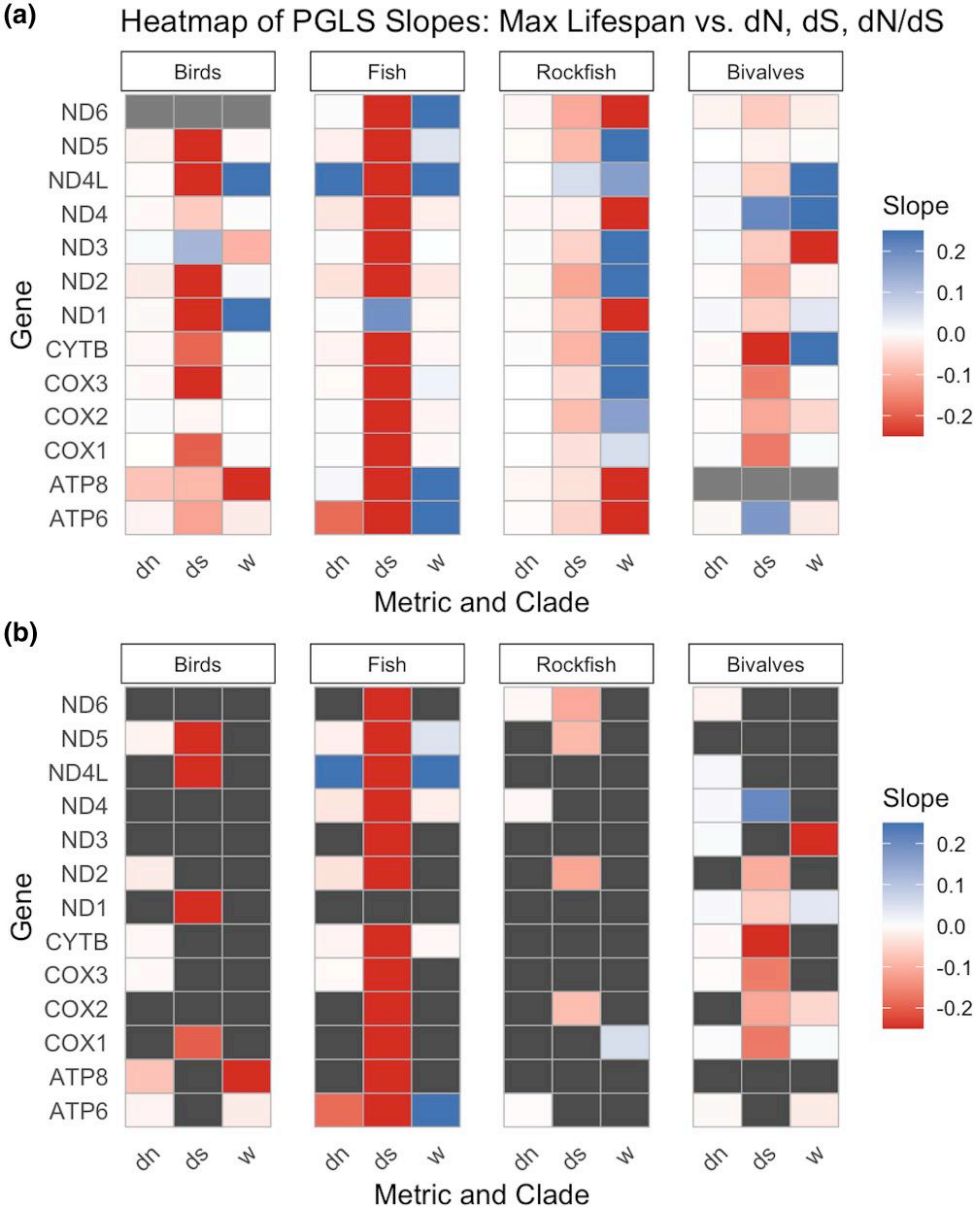
Correlation heatmap of mitochondrial evolutionary metrics and maximum lifespan across four clades. a) Heatmap displaying the PGLS slope estimates between maximum lifespan and mitochondrial evolutionary metrics (dN, dS, and dN/dS) across 13 mitochondrial genes in four clades (birds, fish, rockfish, and bivalves). Positive slopes are represented in blue, and negative slopes in red, with missing values in gray (*ATP8* in bivalves and *ND6* in birds). b) Opaque cells indicate statistically significant relationships (at *P* < 0.05), while solid gray cells display nonsignificant trends.

To determine if particular mitochondrial genes were heavily influencing these trends, an additional correlation was tested individually for each of the 13 mitochondrial-encoded genes using divergence time + PGLS corrected dN and dS (Fig. 3). We focused on the divergence time + PGLS model for gene-specific analyses, as it most closely reflected the overall trends observed across normalization methods while offering stronger explanatory power. For all vertebrate clades, the majority of individual genes showed a negative correlation between max lifespan and both dN and dS. For fish, 62% of the genes had negative correlations for dN, and 92% were negative for dS. For birds, 83% of genes had negative correlations for dN, and 92% were negative for dS. For rockfish, 77% of the genes had negative correlations for dN, and 92% were negative for dS. For bivalves, dS followed the same trend as with the vertebrates—a majority of genes possessed negative correlations (83% of genes). However, this trend vanished with dN values within bivalves, with an even split of positive (50%) and negative (50%) correlations across the 12 genes. When analyzed independently, several genes showed significant negative correlations between substitution rates and maximum lifespan in each dataset, but only dN in *ND4L* in fishes showed a statistically significant positive correlation (Fig. 3). Overall, these results indicate a consistent trend across the mitochondrial genome for each of the clades, with no one gene driving the observed trends. A breakdown of the values used to generate Fig. 2 can be found in Table S1.

### Inconsistent Selection on mtDNA in Longest-Lived Species

The dN/dS ratio (indicative of selection pressure) showed a significant positive relationship with lifespan for the fish clade (*P* < 0.001, *r*^2^ = 0.40) and a significant negative correlation in the rockfish clade (*P* < 0.001, *r*^2^ = 0.40) for the concatenated set of mitochondrial genes when using PGLS models (Fig. 1c and d) (Fig. 2c and d). The dN/dS ratios for the individual genes for both of these clades are fairly evenly split between positive and negative correlations between dN/dS and lifespan (Fig. 3). For the bird and bivalve concatenated mtDNA data, there was no significant correlation between dN/dS and max lifespan (*P* ≥ 0.4, *r*^2^ ≤ 0.07 for both clades) (Fig. 1a and b) (Fig. 2a and b). The individual gene analysis showed similar, inconsistent, or nonsignificant correlations for birds, although for bivalves several independent genes showed significant, negative correlations between dN/dS and lifespan (Fig. 3).

**Fig. 4.**
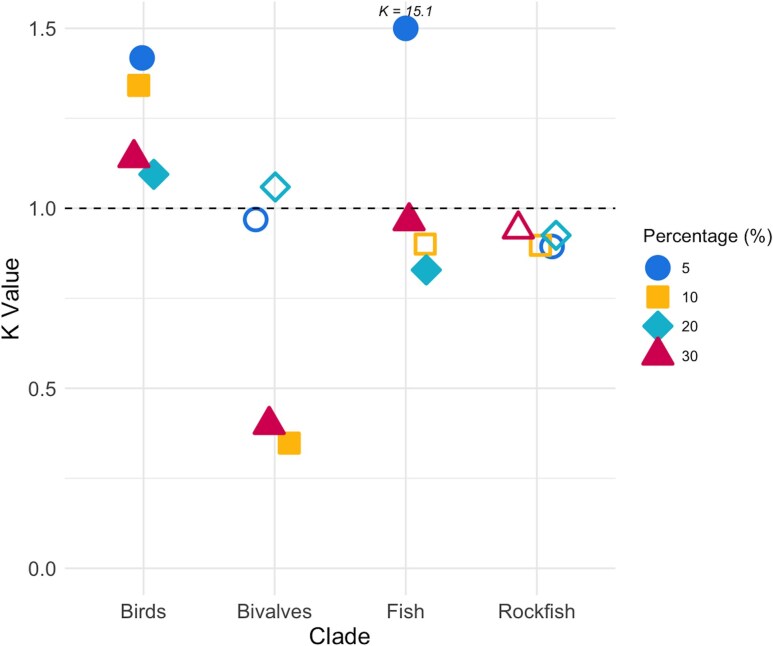
RELAX analysis of mtDNA selection pressures across clades. The plot displays *K* values obtained from RELAX analyses of mitochondrial genomes in four clades (birds, fish, rockfish, and bivalves) at four sensitivity thresholds (test branches corresponded to species with top 5%, 10%, 20%, and 30% of maximum lifespan in each clade). Solid shapes indicate *P* < 0.05, while open shapes indicate *P* > 0.05. The dashed line at *K* = 1 marks the threshold for neutral selection, with *K* values above 1 indicating intensified selection and values below 1 indicating relaxed selection.

Although dN/dS ratios were not generally correlated with lifespan, we tested whether the concatenated set of mt genes was generally under relaxed or positive selection in species with the longest lifespans using RELAX (Wertheim et al. 2015). For birds we found intensified, positive selection in the top 30% longest-lived species (*k* = 1.12, *P* < 0.001), which was generally supported when using other subsets of the data (Fig. 4). For fishes, intensified selection was detected in the top 5% of longest-lived species (*k* = 15.11, *P* < 0.001), but this trend was nonsignificant or reversed (with long-lived species showing relaxed selection) when examining other data subsets (top 10%, 20%, and 30% longest-lived species) (Fig. 4). For rockfish, there was no evidence of intensifying or relaxed selection for any of the datasets analyzed (*P* > 0.05, 0.84 < *k* < 0.98 for all data subsets, Fig. 4). For bivalves, there was evidence for intensifying selection in the top 5% (*k* = 1.12, *P* = 1) and top 20% (*k* = 1.12, *P* = 0.56) of longest-lived species. However, selection was relaxed in the top 10% and 30% of longest-lived bivalves (*k* = 0.35, *P* < 0.001 for both data subsets, Fig. 4).

### Lifespan Has Minimal Effects on Mutation Spectra, with Avian Exceptions

**Fig. 5.**
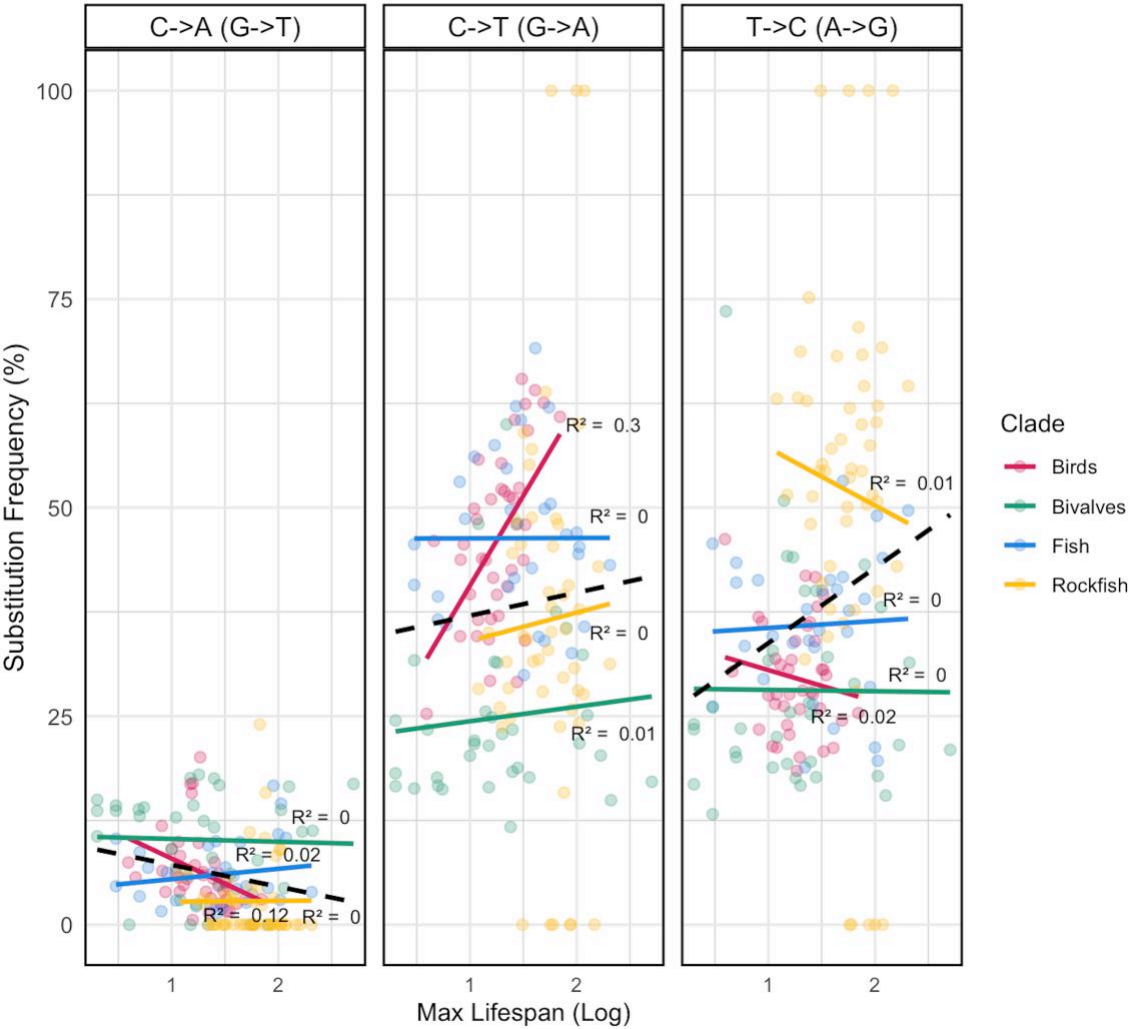
Correlation between substitution frequency and Maximum lifespan across four clades. The figure shows the correlation between the frequency of three substitution types (C→T/G→A, T→C/A→G, and C→A/G→T) and maximum lifespan across birds, fish, bivalves, and rockfish. Mutation frequency is plotted against the log-transformed maximum lifespan for each clade, with linear regression lines highlighting trends. The dashed line is the combination of all clades.

Across all four clades, the correlations between specific proportions of substitutions and lifespan were largely statistically nonsignificant, with no significant trends detected in rockfish, bivalves, or fish species (*P* > 0.05 for all comparisons, Fig. 5). In other words, long-lived species didn’t show a particularly low or high fraction of C→A, C→T, or T→C substitutions in these datasets compared to short-lived species. However, in birds, the proportion of substitutions associated with replication errors (C→T) was positively correlated with lifespan (*P* = 1.8 × 10^−4^, *r*^2^ = 0.3), and the proportion of substitutions associated with polymerase misincorporation events (T→C) was negatively correlated with lifespan (*P* = 0.48, *r*^2^ = 0.12). No significant trends were observed for T→C substitutions (associated with replication errors and polymerase misincorporation events) in any clade.

### Lifespan Explains Substitution Rate Variation Independently of Other Life History Traits

**Fig. 6.**
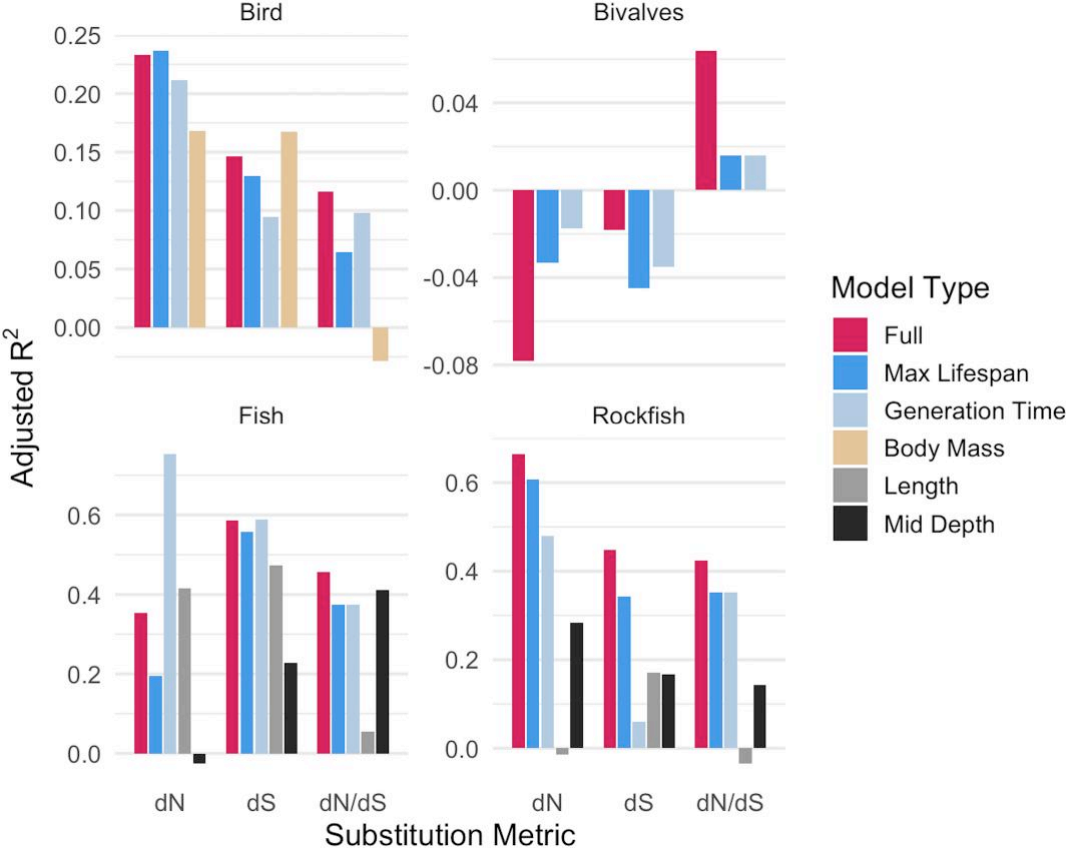
Maximum lifespan independently explains variation in mitochondrial substitution rates across clades. The figure shows adjusted *r*^2^ values from phylogenetic generalized least squares (PGLS) models comparing the explanatory power of all life history/ecological covariates combined (multivariable PGLS model, labeled as “full model” bar in figure) versus single-variable PGLS models. All models were performed on divergence time–normalized substitution metrics (dN, dS, and dN/dS) and assumed Brownian motion evolution (*λ* = 1). VIF results show that collinearity is low between maximum lifespan and the other variables (Fig. S2). Negative adjusted *r*^2^ values (observed in some bivalve models) indicate that a predictor explains less variation than an intercept-only model; this does not imply model misspecification, but rather reflects weak explanatory power relative to sample size and effect magnitude.

To evaluate whether the observed relationships between maximum lifespan and molecular evolutionary rates were confounded by other life history or ecological traits, we conducted both single-variable PGLS analysis and multivariable PGLS analyses. Nearly all confounding variables (ie generation time, body mass, maximum length, and depth) significantly correlated with concatenated mtDNA CDS dN, dS, and dN/dS (corrected for divergence time) during single-variable PGLS analysis for all vertebrate clades (Fig. 6, Table S1). However, subtractive PGLS models revealed that removing individual predictors—including lifespan—from the full model had minimal impact on adjusted *r*^2^, indicating substantial overlap in explanatory power among life history traits (Fig. S1). For bivalves, generation time (the only confounding variable included for this dataset) explained little or no variance for dN, dS, or dN/dS. Despite this overlap, single-variable PGLS models showed that lifespan alone explained a substantial proportion of variation in dN, dS, and dN/dS across all vertebrate clades, often approaching the explanatory power of the full multivariable model (Fig. 6).

To assess whether lifespan was confounded by other life history traits in the multivariable models, we calculated variance inflation factors (VIF) for all predictors within each clade. Across all datasets, VIF values remained low (all < 3), indicating minimal collinearity among covariates (Fig. S3). Additionally, pairwise Pearson correlation matrices showed that although some life history traits were moderately correlated (eg lifespan and generation time; *r* ≈ 0.3 to 0.6), most correlations were weak (*r* < 0.3), and no trait pair consistently exceeded the strong-correlation threshold (*r* > 0.8) across clades (Fig. S4). Together, these results indicate that while life history predictors share overlapping explanatory power, lifespan captures a distinct and biologically meaningful dimension of substitution rate variation that is not reducible to any single covariate.

## Discussion

### Lifespan Explains Mitochondrial Mutation Rates in Vertebrates, but Not Selection

We found a strong negative correlation between mitochondrial substitution rates and max lifespan that persists across three vertebrate clades under most normalization approaches (Fig. 1). This trend was consistent across divergence time–normalized models but differed under generation time normalization, which in some cases produced positive correlations between lifespan and substitution metrics. The negative relationship observed under divergence time normalization was consistent across all vertebrate clades analyzed, including a broad sampling of fishes—a group that hasn’t previously been studied in this context (although see Cam et al. 2024). These findings are consistent with those observed in past studies on mammalian and bird mtDNA (Nabholz et al. 2007, 2009). These past studies used divergence time to normalize rates of evolution along with phylogenetic correction, and were conducted on a single mitochondrial gene (cytochrome b) (Nabholz et al. 2007, 2009). Our results support that this trend extends across the whole mtDNA, although statistical significance varied among individual genes (Fig. 3), possibly due to variation in statistical power among genes of different lengths (Havird and Santos 2014). This idea that lifespan is tightly linked to mutation rate variation is supported by independent comparative work using nuclear DNA across mammals (Cagan et al. 2022).

A past study on rockfish mtDNA substitution rates (looking at two protein-coding and five RNA mtDNA genes) also shows a negative correlation between rates and lifespan, although it was only statistically significant for dS (Hua et al. 2015). However, that analysis was performed using dN and dS values without correction for divergence time or phylogenetic relationships. In our dataset, normalizing substitution rate for divergence time intensified the negative correlation in rockfishes, producing statistically significant trends in dN as well as dS and a great increase in the amount of variance explained (Fig. 1). These results further highlight the importance of normalizing rates of evolution using standard methods.

In contrast to vertebrate clades, we found no evidence of a correlation between lifespan and mitochondrial substitution rates in bivalves (Fig. 1). These results contradict past findings that show a negative correlation between lifespan and dS in bivalve mtDNA (Mortz et al. 2021). However, this study also did not normalize substitution rates for divergence time or phylogeny. When applying the same non-normalization approach to the species in our study, we also found that dS and dN are negatively correlated with lifespan, although not significantly (Fig. 1). However, in every other model—including the more thorough divergence time + phylogeny corrected models—dS and dN were positively correlated with lifespan, although still not significantly (Fig. 1). Overall, our results suggest that the dynamics underlying mitochondrial evolution and lifespan may differ between vertebrates and invertebrates.

For vertebrates, the consistency of trends across both nonsynonymous and synonymous substitutions (Fig. 1 and 2) suggests that the primary driver of lower dN and dS in long-lived vertebrates is lower mtDNA mutation rates and not variation in selection (Fig. 4). The consistency of trends across genes (Fig. 4) also suggests that mutation mechanisms overall, not selection for particular mitochondrial processes, may be driving these trends. Supporting this, dN/dS (an indicator of selective pressure) showed largely inconsistent trends with lifespan (Fig. 1 and 2). Similarly, RELAX results suggested an inconsistent selection pressure on mtDNA in long-lived species overall, although positive selection in the most long-lived species of some clades. However, we caution that such molecular signatures of selection in mtDNA are prone to overinterpretation (Zwonitzer et al. 2023; Iverson et al. 2025).

Lastly, do biological or statistical processes explain the observed negative relationship between mtDNA substitution rates and lifespan? We cautiously favor a biological explanation. Lifespan consistently explained substitution rate variation (normalized to divergence time, see below for discussion on generation time effects) even after accounting for potentially correlated traits such as body size and environmental depth (Fig. 6), and variance inflation analyses confirmed minimal collinearity among predictors (Fig. S3; Fig. S4). However, other life history or demographic variables may also dictate mitochondrial substitution rates—such as effective population size (Woolfit and Bromham 2005), reproductive strategy (Romiguier et al. 2014), or sex-specific factors like sex-biased inheritance or skewed sex ratios that are particularly relevant to mtDNA evolution (Gemmell et al. 2004; Havird et al. 2019).

If biological processes underlie this trend, we expected specific mutation pathways might be implicated. However, at the resolution of our analyses, this also did not seem to be the case, as substitution spectra (which may approximate underlying mutation spectra) showed no evidence of selective suppression of specific mutational classes (Fig. 5). Thus, the observed reduction in substitution rate appears broadly similar across mutation types. Notably, G→T substitutions, a hallmark of ROS-induced damage, were not strongly correlated with lifespan, which contradicts predictions under the mitochondrial free radical theory of aging (MFRTA). Nevertheless, the apparent uniformity of substitution spectra should be interpreted with caution. Comparative substitution rate analyses may lack the resolution needed to detect more subtle, process-specific mutational biases, particularly if these effects are weak or obscured by evolutionary averaging across lineages with deep divergences. Recent work suggests that fine-scale mutational asymmetries can persist even when overall substitution spectra appear broadly similar, including differential contributions from POLG-mediated replication errors versus ROS-associated damage (Mikhailova et al. 2022; Iliushchenko et al. 2025). Under this view, one plausible hypothesis is that POLG-associated symmetric mutations are preferentially reduced in long-lived species, whereas ROS-associated asymmetric mutations scale differently—an idea that will require direct experimental validation. These patterns suggest that the relationship between lifespan and mitochondrial substitution rates reflects a net reduction in mutational input, potentially influenced by factors such as slower mitochondrial turnover/replication or generation time in general, while allowing for mechanistic differences at finer mutational scales, that cannot be fully explained by classical life history scaling or oxidative damage models (Popadin et al. 2007; Nabholz et al. 2009; Shen et al. 2010).

Overall, these results highlight the value of taking a broad comparative approach to understanding mitochondrial evolution and aging. Across clades, long-lived species tend to accumulate fewer mitochondrial substitutions, but bivalves did not show this pattern when using a more thorough normalization method. Although the cause of this trend is still largely unknown, clade-specific biology may play an important role. For example, different animal groups use different types of mtDNA mutation repair mechanisms (Pont-Kingdon et al. 1995; Muthye and Lavrov 2021), which may explain some variation in how substitution rates vary with lifespan. Expanding this framework to other groups, especially invertebrates with unusual or regenerative life histories, could help clarify what forces are driving mitochondrial genome stability across the tree of life.

### Normalization Methods Drastically Change the Interpretation of Lifespan–Substitution Rate Relationships

Characterizing substitution rates across a phylogeny has been used to analyze rates of evolution for decades and correlate them with various ecological or life history traits (Zuckerkandl and Pauling 1965; Kimura 1968; Welch et al. 2008; Nabholz et al. 2009; Lanfear et al. 2010; Thomas et al. 2010). However, interpreting these rates is not straightforward, with wide variation among studies. Most commonly, “raw” dN and dS values are used “as is” because these rates are already normalized to the number of possible synonymous or nonsynonymous changes within a sequence based on the genetic code. However, dN and dS rates are relative to a branch within a phylogeny (eg the terminal branches leading to extant species in our analyses), and the length of these branches in absolute divergence times can vary dramatically. For example, within our bivalve clade, terminal branch lengths ranged from 500 million to 2 million years. Substitution rates will always appear higher on longer branch lengths, regardless of what metric is being tested, which can lead to spurious conclusions. For example, by using raw dN and dS values, we might have concluded that longer-lived species had lower substitution rates if longer-lived species happened to diverge more recently than short-lived species in a given dataset.

To solve this issue, many studies normalize substitution rates for divergence time, which is inherently a more meaningful metric where substitution rates are normalized to the number of relevant sites in an alignment and the amount of time where changes could have occurred. For example, when “raw” dN and dS values were calculated for our datasets, rockfishes show an order of magnitude lower values because this clade is ∼10 times younger than the other clades we examined. By using the “raw” dN and dS values for rockfish along with other fishes, we would have concluded that the long lifespans of rockfishes in general were associated with low mtDNA substitution rates, but just because rockfish are relatively recently diverged. Using “raw” values is only appropriate when divergence times do not correlate with the trait of interest (eg lifespan), which is troublesome to assume a priori.

Additionally, many previous correlative studies do not correct their data for phylogeny. This is important because data obtained from across a phylogeny are not independent (Symonds and Blomberg 2014), and this independence should be corrected for. In our study, we might expect more closely related taxa to have more similar dN or dS values and/or similar lifespans if changes to mtDNA replication machinery or ROS scavenging enzymes occurred in their common ancestor. Therefore, we primarily reported trends that were corrected for phylogenetic relationships through the use of PGLS. When our data were corrected for divergence times, but not phylogeny (Fig. 1), the story changed, with some trends remaining similar, but significance and variance generally intensifying with the implementation of phylogenic normalization. Again, this emphasizes that the choice of methodology matters when correlating evolutionary rates with other factors.

Although very seldom considered in previous similar studies, generation time can also drastically influence rates of molecular evolution. Animals with shorter generation times will experience more generations, and therefore the germline will likely experience more rounds of cell division across the same absolute time span. This may be especially important for mtDNA, which is often kept in a “quiescent” state in egg cells that are not dividing (Marescal and Cheeseman 2020; Van Der Reest et al. 2021; Hocaoglu and Sieber 2023). Not surprisingly, studies attempting to quantify absolute rates of mtDNA mutation normalize rates on a per-generation basis (Denver et al. 2000). In most prior evolutionary studies correlating max lifespan with mitochondrial dS and dN, generation time is simply included as a covariate in models that already normalize substitution rates by divergence time (Nabholz et al. 2007, 2009). While that approach is informative, it may underestimate generation time's true influence. To better capture its effect, we attempted to normalize dN and dS to generation time, generating rates of dN and dS per 100,000 generations (Fig. 1; Fig. S1). Generation times were based on those in extant taxa, which may be an oversimplification, as generation times can change drastically along a phylogenetic branch over millions of years (Lynch et al. 2023). Once this normalization was applied, our results again changed drastically, with the previously negative correlations with lifespan reversed in both birds and fish (Fig. 1; Fig. S1).

However, this shift in correlation direction when normalizing by generation time does not invalidate the original finding that long-lived species tend to have lower substitution rates. Instead, it reframes its interpretation. The divergence time–normalized model suggests that long-lived species accumulate fewer mutations over evolutionary time. But the generation time–normalized model reveals that when mutations are considered per-generation, long-lived species actually experience more substitutions per generation than short-lived ones. This suggests that the observed reduction in substitution rate may be less about selection to preserve mtDNA in long-lived species and more about the cumulative effect of fewer generational turnovers. This distinction is critical. If increased lifespan itself reduced mutation rates (as posited by the mitochondrial theory of aging), we would expect the negative trend to persist or even strengthen under per-generation normalization. The fact that it reverses in birds and fish instead suggests generation time is a stronger predictor than lifespan for most clades (although the two are highly correlated).

The exception is rockfish. In this group, the negative correlation between lifespan and substitution rates persists even after generation time normalization, meaning long-lived species experience significantly fewer substitutions per generation than short-lived counterparts. This supports a more direct link between lifespan and mtDNA stability. Notably, rockfish fecundity increases with age and size (Berkeley et al. 2004), which allows older individuals to contribute disproportionately to reproduction. In such a life history strategy, the selective pressure to maintain mitochondrial integrity with age may be much stronger. This clade-specific contrast highlights that while generation time broadly explains substitution rate variation in vertebrates, true selection on lifespan-linked mutational suppression may operate only in taxa where longevity directly enhances fitness.

These results call into question previous studies that have reported negative correlations between lifespan and substitution rates without thoroughly considering the influence of various normalization methods (eg Nabholz et al. 2007, 2009; Welch et al. 2008; Mortz et al. 2021). Our findings suggest that these correlations may reflect statistical artifacts deriving from differences in generation time, phylogenetic signal of evolutionary rates, and/or divergence times. Future research should explicitly test how different normalization approaches affect evolutionary rate correlations, particularly when comparing across taxa with diverse reproductive strategies and life histories.

## Materials and Methods

### Dataset Selection

To explore the relationship between mtDNA evolution and longevity, publicly available mitochondrial genome sequences were gathered for analysis. The species selected spanned four major clades: birds (class Aves, *n* = 40), bony fishes (class Actinopterygii, *n* = 30), bivalves (class Bivalvia, *n* = 35), and rockfish (family Sebastidae, *n* = 51). We focused on these clades given the availability of complete mtDNAs, variability in lifespan across species, and previous studies suggesting a relationship between mtDNA substitution rates and lifespan (Nabholz et al. 2009; Kolora et al. 2021; Mortz et al. 2021). We included rockfish, a relatively young clade (originating ∼15 million years ago) within Actinopterygii, because their remarkable lifespan variation—ranging from 2 years to over 200 years—makes them a unique system for studying mitochondrial evolution and longevity (Hua et al. 2015; Kolora et al. 2021). Each species included in the study had to meet two inclusion criteria: an accessible complete mitochondrial genome sequence and a documented maximum recorded lifespan (sourced from governmental databases, curated publications, or reputable biological repositories; see Table S1). We also gathered several other life history traits known to vary with lifespan when possible (Table S1). Rockfish mitogenomes were obtained from https://github.com/evolgen/rockfishgenomeproject/tree/main/assembly_annotation/mitogenome/ and are associated with recent genomic resources from rockfishes (Kolora et al. 2021). Mitogenomes for the remaining clades were collected from GenBank (see Table S1 for accession numbers). Custom scripts were used to download, parse, align, and process the 13 protein-coding genes from mitogenomes (https://github.com/thekzwon/mito_accessions_to_full_alignment).

Known species relationships for each clade were taken from existing studies based on genomic datasets for the birds and bony fish datasets (Betancur et al. 2017; Kimball et al. 2019). For species missing from these reference trees, inferences were made based on topology using additional phylogenies for birds (Barker et al. 2004; Wink et al. 2009) and bony fishes (Westneat and Alfaro 2005; Craig and Hastings 2007; Hyde and Vetter 2007; Wang et al. 2012; Santos et al. 2013; Shedko et al. 2013; Arce-H et al. 2017; Bagley et al. 2018; Grande et al. 2018; Shen et al. 2020). For the rockfish and bivalve datasets, new phylogenies were inferred from mitochondrial genome coding sequences. For rockfishes, a maximum likelihood phylogeny was inferred using nucleotide sequences based on the aligned, concatenated 13 mitochondrial protein-coding genes as input into RAxML-NG BlackBox (Kozlov et al. 2019) under the default substitution model GTR + G. For bivalves, the phylogeny was constructed using aligned amino acid sequences from 12 concatenated protein-coding genes (excluding *ATP8* due to frequent annotation inconsistencies in some bivalve lineages; Lubośny et al. 2018). The final bivalve tree was inferred using RAxML and default settings as implemented in Geneious Prime (Anon) and cross-referenced with a published mtDNA phylogeny for bivalves (Mortz et al. 2021) to ensure topological consistency.

### Calculating Mitochondrial Substitution Rates

To quantify nonsynonymous (dN) and synonymous (dS) substitution rates across species in each of the four datasets, the CODEML module of PAML was used (Yang 2007). This module was used to determine dN and dS for both the concatenated set of mitochondrial protein-coding genes as well as for each gene individually (excluding *ND6* from birds and ATP8 in bivalves due to missing data). The free-ratio model (Model 1) was implemented in CODEML, which allows dN/dS to vary across each phylogenetic branch, providing lineage-specific estimates of selection pressure. Because our analyses required species-specific dN and dS values, we utilized the branch model to capture variation in metrics of interest across the phylogeny. We used branch-specific dS and dN values for each of the terminal branches in each dataset, which corresponded to a species with a known maximum lifespan, and ignored values for the internal branches.

In total, substitution rates (dN and dS) were analyzed in five ways: unnormalized (raw rates), normalized for divergence time, normalized for generation time, normalized for divergence time with phylogenetic correction, and normalized for generation time with phylogenetic correction. To normalize for divergence time (and thus account for the impact of branch length), each of the four phylogenies was fossil-calibrated using the Chronos function in ape (Paradis et al. 2004). Fossil divergence estimates were obtained from TimeTree (Kumar et al. 2022) and primary literature, selecting calibration nodes based on well-supported clade divergences. Minimum and maximum age estimates were assigned based on fossil records or secondary molecular clock estimates when direct evidence was unavailable (see Table S1). Trees were visualized and annotated in IcyTree (Vaughan 2017), with node labels assigned to match calibration points. Chronos (Vrahatis et al. 2016) was implemented using a relaxed molecular clock (*λ* = 1) to estimate divergence times, which were then used to normalize dN and dS rates (as dN or dS per million years of evolution) across species. To normalize for generation time, the total number of generations since divergence (using species-specific generation time estimates and estimated divergence times) was calculated, and then dN and dS values were expressed as rates per 100,000 generations. For details on phylogenetic corrections, see the “Statistical Analyses” section.

### Selection on mtDNA in Long- vs. Short-Lived Species

The dN/dS ratio was also calculated via codeml to examine selection in mt protein-coding genes. In contrast to substitution rates, dN/dS values in this study were only analyzed as either “raw” or phylogenetically corrected (as the ratio is inherently normalized for divergence time). To determine whether differences in dN/dS ratios across species were due to relaxed or intensified selection, we ran RELAX analyses (Wertheim et al. 2015) using the Datamonkey web server. RELAX estimates a selection intensity parameter (*k*) based on the distribution of dN/dS ratios across sites in a set of test versus reference branches in a phylogeny, where *k* > 1 indicates intensified selection, *k* < 1 suggests relaxed selection, and *k* = 1 represents no change in selection pressure (Wertheim et al. 2015). Statistical significance is assessed using likelihood ratio tests comparing a model where dN/dS classes are the same between reference and test branches. Separate sets of RELAX analyses were conducted for each clade, using concatenated mtDNA sequences. RELAX requires the specification of reference and test branches across a given phylogeny (Wertheim et al. 2015). For this study, reference branches were designated as short-lived species, while the test branches were defined as long-lived species. Internal branches were left unassigned. Given that RELAX requires branch assignments, we conducted four separate analyses per clade, varying the threshold for long-lived species to the top 5%, 10%, 20%, and 30% of maximum lifespan observed within a dataset. By testing multiple longevity thresholds, we aimed to minimize bias and ensure a comprehensive assessment of selection intensity across species.

### Substitution Spectra Analysis

Beyond examining substitution rates and selection, we generated substitution spectra for each species, specifically focusing on three abundant, biologically relevant substitution types: C→A (coded the same as G→T in our analyses), C→T, and T→C. These mutation types were selected based on both previous literature and our own findings, as they represent the most frequent mitochondrial DNA substitutions (Duncan and Miller 1980; Nordmann et al. 1988; Spelbrink et al. 2000; Longley et al. 2001; Song et al. 2005; Zheng et al. 2006). Each of these mutations is strongly linked to a relevant source thought to drive mtDNA mutations: C→A mutations are characteristic of ROS-induced oxidative damage, C→T mutations result from spontaneous deamination or replication errors, and T→C mutations are specific to replication errors.

We used custom scripts to generate a substitution spectrum for each species in each dataset (available via GitHub: https://github.com/thekzwon/Metazoa_mtDNA_substitution_spectra). Briefly, ancestral sequences were inferred for each node in the phylogeny using MEGA11 (Tamura et al. 2021), and all substitutions leading to each terminal branch were considered. To account for compositional biases, substitution proportions were normalized by the nucleotide composition of the reconstructed ancestral sequence. This approach allowed us to express the final substitution spectra as the percentage of each substitution type for each species, controlling for underlying sequence composition. With these substitution spectra, we asked whether specific substitution types disproportionately occurred in species with shorter lifespans (eg are short-lived species more affected by ROS and show a higher proportion of C→A substitutions, as predicted under the MFRTA). To assess whether substitution spectrum frequencies were associated with lifespan, we performed Pearson correlation tests between the relative frequency of each mutation type and maximum lifespan across all species for each of the four clades.

### Statistical Analyses

Our study took a correlational approach, examining how mtDNA evolution may relate to maximum lifespan. To investigate the relationship between molecular evolution rates (dN, dS, and dN/dS) and maximum lifespan, we employed several normalization approaches. First, raw rates (for dN and dS, dN/dS is already internally normalized) as estimated by CODEML were correlated with lifespan. Next, rates normalized to divergence time or number of generations were correlated with lifespan (see above). Finally, phylogenetic generalized least squares (PGLS) modeling (Grafen 1989; Symonds and Blomberg 2014) using the *caper* and *nlme* packages in R was used to correlate both types of normalized rates with lifespan while controlling for phylogeny. For each of the four clades, a separate single-predictor PGLS analysis was performed correlating maximum lifespan with dN (corrected for divergence time), dS (corrected for divergence time), or dN/dS. Preliminary analyses showed that Pagel's lambda (*λ*) optimization was unstable, so all models were run using Brownian motion (*λ* = 1), which assumes a random walk model of trait evolution. In another set of analyses, we used known generation times for each species (Table S1) coupled with estimated divergence times to correct dN and dS for the number of generations (eg dS per 100,000 generations) instead of absolute divergence times in millions of years.

To determine the impact of other ecological factors that may also be driving lifespan, additional single-predictor PGLS models were run. This was conducted because underlying ecological factors may drive trends in both mtDNA substitutions and lifespan (eg fish that live in deeper waters generally live longer and may have slower substitution rates; Cailliet et al. 2001). For all four clades, we first conducted single-predictor PGLS models to assess the independent correlations between dN, dS, or dN/dS and generation time, as generation time tends to have a strong correlation with rates of molecular evolution across vertebrates and invertebrates (Denver et al. 2000). Additional single-predictor PGLS models were also run for three of the four clades. For birds, one model was performed that included average adult body mass (g). For the fish and rockfish clades, additional models included max body length (cm), minimum depth (m), maximum depth (m), and midpoint depth (m). To maximize the sample size for each analysis, we created separate datasets for each predictor, ensuring that species were only excluded when missing data for the specific predictor of interest.

To determine whether relationships between dN, dS, or dN/dS and maximum lifespan persist when accounting for these potential confounding variables, multivariable PGLS models were conducted. Specifically, we implemented a subtractive model selection approach. Briefly, a full model including all covariates was constructed, and predictors were individually removed to evaluate their unique contribution to model performance. This allowed us to assess the degree to which each variable independently influenced explanatory power, including variables such as generation time, body mass, maximum length, and depth. Model performance and variable influence were assessed through changes in adjusted *r*^2^ and statistical significance. All continuous predictors were log_10_ transformed before analysis to normalize distributions and enhance interpretability. To assess potential collinearity among variables, we calculated pairwise correlations and variance inflation factors (VIF) within each clade. This ensured model estimates were not confounded by strongly correlated variables (Zuur et al. 2010).

## Supplementary Material

evag067_Supplementary_Data

## Data Availability

All processed datasets underlying the results are provided as a single supplementary data file. No new custom software was developed; analyses used existing packages as described in Materials and Methods.
