# Supplementary material for: Lifespan Predicts Mitochondrial Substitution Rates across Vertebrates, but Methodology Matters
Source: Genome Biol Evol. 2026 Mar 16;18(3):evag067. doi: 10.1093/gbe/evag067 (PMC13034128; doi:10.1093/gbe/evag067)
Supplement: evag067_Supplementary_Data [file evag067_supplementary_data.zip › Supplement_Figures.pdf]

Supplement Figure 1: Correlation between Max Lifespan and dN, dS, and dN/dS Normalized to 100,000 Generations Across Four Clades

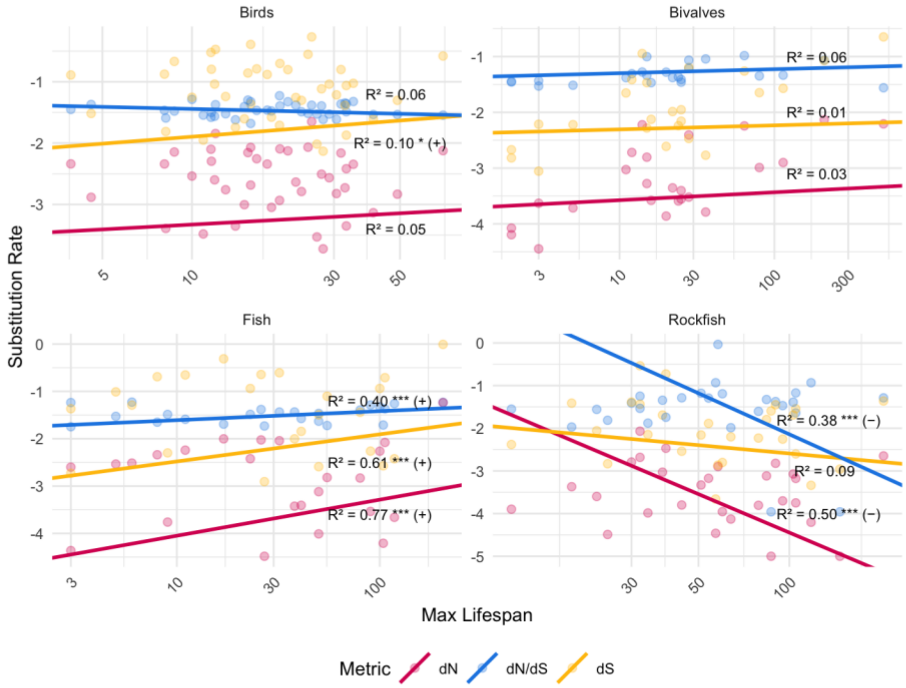

Relationships between maximum lifespan (X-axis, log scale) and three mtDNA rates of evolution (Y-axis, log scale): dN, dS, and dN/dS for three datasets (birds, fish, rockfish, and bivalves). The dN and dS metrics values are per 100,000 generations and corrected for phylogeny. dN/dS is only corrected for phylogeny using PGLS. Solid lines represent PGLS regression trends (phylogenetically corrected), with associated R^2 values shown. Direction of trend (+ or –) and statistical significance is also shown (*, **, *** = $P < 0.05$, 0.01, and 0.001, respectively).

Supplementary Figure 2: Most life-history traits uniquely contribute to mitochondrial substitution rate variation across clades.

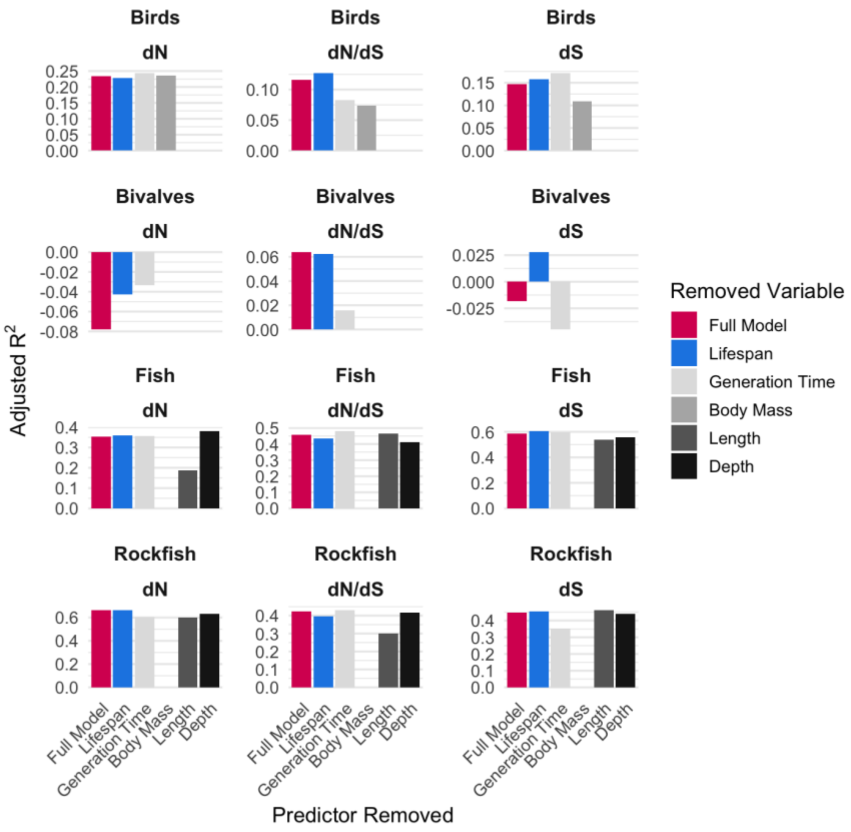

Adjusted R² values from subtractive phylogenetic generalized least squares (PGLS) models in which individual life-history or ecological predictors were removed from the full additive model. For each clade and substitution metric (dN, dS, dN/dS), the "Full Model" bar reflects the adjusted R² from a model containing all available covariates. The remaining bars represent adjusted R² values from models excluding a single predictor, as indicated on the x-axis. Greater reductions in adjusted R² indicate a stronger individual contribution of the removed trait to the model's explanatory power. All models were run on divergence time–normalized substitution metrics and assumed Brownian motion (λ = 1).

Supplementary Figure 3: Variance Inflation Factor (VIF) values for predictors of maximum lifespan across clades.

References (for Supplementary Figure 3)

Kim JH. 2019. Multicollinearity and misleading statistical results. Korean J Anesthesiol. 72:558–569. doi:10.4097/kja.19087.

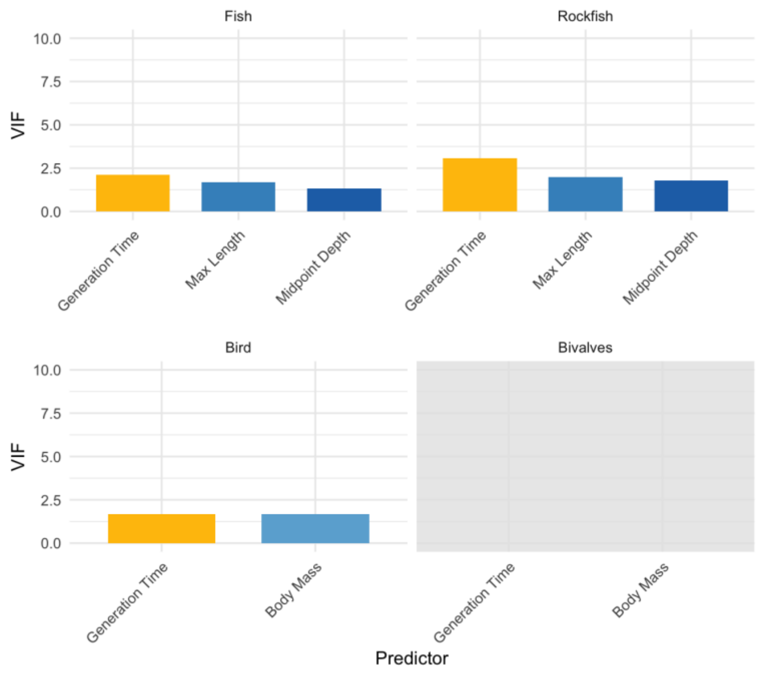

VIF was calculated from linear models regressing Max Lifespan against all other life-history and ecological traits used in the multivariable PGLS analyses (e.g., generation time, body mass, maximum length, depth). A VIF value >5 is typically considered indicative of problematic multicollinearity (Kim, 2019). All traits showed low VIF values (<3), indicating minimal collinearity and suggesting that these variables contribute largely independent explanatory power in predicting lifespan-associated patterns in molecular evolutionary rates. Bivalves were excluded from this analysis because only two predictor variables were available.

Supplementary Figure 4: Correlation matrix heatmap of life-history and ecological traits used in lifespan–substitution rate models

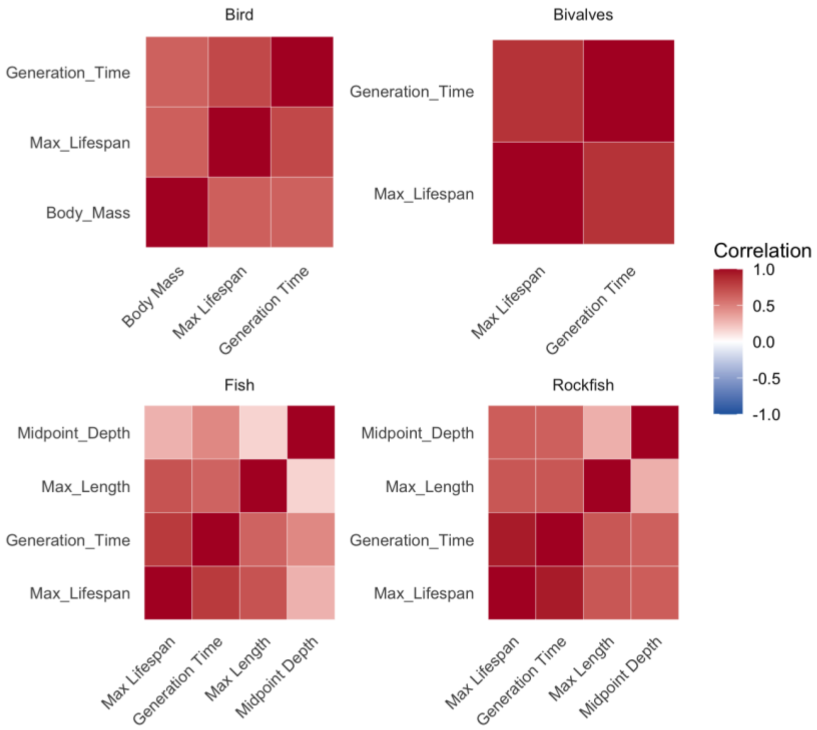

Pearson correlation coefficients between all predictor variables used in the multivariable PGLS models, calculated separately for each clade. This matrix provides an overview of linear relationships among traits, including maximum lifespan, generation time, body mass, maximum length, and depth (where available). Strong correlations (e.g., $r > 0.8$) indicate potential collinearity that may influence model interpretation. This analysis complements the VIF analysis by quantifying pairwise trait associations and helps clarify shared variance among confounders.
